# The acceptability of asking women to delay removal of a long-acting reversible contraceptive to take part in a preconception weight loss programme: a mixed methods study using qualitative and routine data (Plan-it)

**DOI:** 10.1186/s12884-022-05077-0

**Published:** 2022-10-18

**Authors:** Susan Channon, Elinor Coulman, Rebecca Cannings-John, Josie Henley, Mandy Lau, Fiona Lugg-Widger, Heather Strange, Freya Davies, Julia Sanders, Caroline Scherf, Zoe Couzens, Leah Morantz

**Affiliations:** 1 Centre for Trials Research, Cardiff University, Neuadd Meirionnydd, Heath Park, Cardiff, CF14 4YS UK; 2 The Welsh Centre for Primary and Emergency Care Research (PRIME), Division of Population Medicine, Cardiff University, 8th floor, Neuadd Meirionnydd, Cardiff, CF14 4YS UK; 3 School of Healthcare Sciences, College of Biomedical and Life Sciences, Cardiff University, Ty Dewi Sant, Cardiff, CF14 4XN UK; 4 Department of Sexual Health, Cardiff and Vale University Health Board, Cardiff Royal Infirmary, Newport Road, Cardiff, CF24 0SZ UK; 5 Health Protection, Public Health Wales NHS Trust, Public Health Wales, 4th Floor Number 2 Capital Quarter, Tyndall Street, Cardiff, CF10 4BZ UK; 6 Independent Patient Representative, Cardiff, UK

**Keywords:** Preconception, Obesity, Weight loss, Long-acting reversible contraceptives, Routine data, Contraception

## Abstract

**Background:**

Having a body mass index (BMI) which is classified as overweight (BMI ≥ 25) or obese (BMI ≥ 30) increases the risk of complications during pregnancy and labour. Weight-management interventions which target excess gestational weight gain during pregnancy have had limited success. Women who use long-acting reversible contraception (LARC) are in contact with services as part of their preparation for conception, creating a potential opportunity to offer a preconception weight-loss intervention.

The aims of this mixed methods study were to assess the acceptability and practicability of a weight-loss intervention which asked people to delay LARC removal in order to lose weight before conceiving.

**Methods:**

Routine UK NHS data were analysed to identify pathways from LARC removal to pregnancy. Qualitative surveys and advisory group discussions with service providers and LARC users with experience of being overweight were conducted and analysed thematically.

**Results:**

Three hundred fifteen thousand seven hundred fifty-five UK women aged 16–48 years between 2009–2018 had at least one LARC-related event (e.g. insertion, removal) and 1.7% of those events were recorded as related to planning a pregnancy. BMI was included in 62% of women’s records, with 54% of those BMI being classified as overweight or obese. Online surveys were completed by 100 healthcare practitioners and 243 LARC users. Stakeholders identified facilitators and barriers associated with the proposed intervention including sensitivities of discussing weight, service-user past experiences, practitioner skills, the setting and ethical implications of the proposed intervention.

**Conclusions:**

Although women and service providers recognised potential benefits, a preconception weight-loss intervention asking people to delay LARC removal posed many barriers, due mainly to the acceptability of such an intervention to women and healthcare practitioners. Weight-loss interventions that target the general population, together with a focus on improving public knowledge of preconception health, may be more acceptable than interventions which solely focus on LARC users. Many of the barriers identified, including communication, understanding and beliefs about weight and risk, appointment systems and the limitations of routine datasets also have relevance for any preconception weight-loss intervention. Work to improve routine datasets and reducing communication barriers to discussing weight are priorities.

**Trial registration:**

ISRCTN14733020 registered 10.05.2019.

**Supplementary Information:**

The online version contains supplementary material available at 10.1186/s12884-022-05077-0.

## Background

BMI rates are important determinants of outcomes in the antenatal, intrapartum and postpartum periods [1, 2] and on child health [2–4]. Although weight management programmes during pregnancy are associated with significant reductions in gestational weight gain (GWG), these reductions had limited impact on associated maternal or child complications [5, 6]. In addition, a meta-analysis investigating the effects of lifestyle interventions on GWG or postpartum weight retention concluded that weight loss prior to pregnancy is probably required to achieve both GWG goals and optimal pregnancy outcomes [6]. With the increasing urgency of tackling this problem driven by rising rates of overweight and obesity [7, 8], attention has turned to preconception health and the potential to reduce obesity prior to conception.

The preconception period has been described as an “underappreciated period in the life course” [9] in terms of its far-reaching impact on maternal and child health in the short and long term [10] and may be considered a “teachable moment” [11] where efforts may be made to positively influence diet and health behaviours. Current NICE guidance states preconception advice should include recommendations regarding a range of health behaviours including folate supplementation and smoking cessation [12]. However, one third of the 600,000 plus live births annually in England and Wales [13] are estimated to be unplanned [14] and few people actively seek a consultation relating to their preconception health (unless there are existing health concerns or uncertainties regarding fertility) [15, 16].

Furthermore, with the exception of a number of ongoing studies [17–22], there has, to date, been limited research examining weight loss interventions (WLIs) that target obesity in the preconception period outside of the very specific context of in-vitro fertilisation [23, 24] and overall the quality of the evidence in this field is very limited [25].

People who use long-acting reversible contraception (LARCs) and require removal of the device to conceive, represent a unique group where there is an opportunity for intervention. A small feasibility study of an intensive WLI offered to women attending for LARC removal, demonstrated that some were willing to consider delaying LARC removal for six months in order to participate [26]. However, high rates of non-participation and attrition from the programme were observed. It has therefore not yet been established what, if anything, the nature of an acceptable intervention involving delayed LARC removal would be.

This study aims to establish if it is acceptable and practicable to conduct a study that asks people who have a body mass index (BMI) which is classified as overweight (BMI ≥ 25) or obese (BMI ≥ 30) to delay removal of LARC to participate in a targeted preconception weight loss intervention. The objectives were:

- 1. to identify the annual number of people of reproductive age (16 to 48 years old) in the UK who request LARC removal and subsequently have a pregnancy.
- 2. to identify the means of identifying people who have a body mass index (BMI) ≥25 at study sites who plan to have LARC removal for the purpose of planning a pregnancy and identify opportunities to intervene.
- 3. to explore the willingness of HCPs to raise weight loss in consultations and recruit eligible women to the proposed WLI.
- 4. to explore the views of people who use/have used LARCs and who self identify as currently or previously having a BMI≥25 as to the acceptability of the proposed WLI.

## Methods

### Design

The study took a concurrent mixed methods approach incorporating use of routine NHS data and qualitative data collection and analysis across two work-packages. Work package 1 (WP1) focussed on establishing the feasibility of defining and understanding the population through routine data, addressing objectives 1 and 2. Work package 2 (WP2) focussed on understanding context and stakeholder views, addressing objectives 3 and 4 (a detailed description of the methods is reported elsewhere [27, 28]. The study was reviewed and approved by the Cardiff University School of Medicine Research Ethics committee on 30^th^ April 2019 (ref 19/42).

### Participants

In WP1 the pre-defined study population identified within the routinely collected datasets were UK women of reproductive age (16 to 48 years inclusive) with at least one consultation identified as LARC-related in the dataset between 1^st^ January 2009 and 31^st^ December 2018.

WP2 included two groups of stakeholders:

i) HCPs who insert and/or remove LARCs, recruited at professional meetings.
ii) Women of reproductive age who self-identify as having/previously having a BMI ≥ 25 and experience of having used a LARC, recruited via a multipoint online recruitment strategy involving advertisements on online spaces including boosted advertisements on Facebook Twitter, Netmums forum, and distribution to potentially eligible participants via Healthwise Wales (a national longitudinal research study and register https://www.healthwisewales.gov.wales/).

### Data collection and analysis

WP1 used data from the Clinical Practice Research Datalink (CPRD), a database of anonymised electronic health records from over 600 primary care practices in the United Kingdom (UK). The CPRD has a coverage of over 11.3 million patients broadly representative of age, sex and ethnicity of of the UK population [29]. The Pregnancy Register lists all pregnancies identified in the CPRD including live births through the CPRD Mother Baby Link [30]. Data access requests to CPRD were reviewed by the Independent Scientific Advisory Committee (ISAC).

Women aged 16 to 48 years were identified using a LARC-related code list (see Additional File 1), pre-defined by the clinical co-investigators, which included insertion, in-situ checks, and removal (referred to in this study as a “LARC event”). These women’s records were then linked to the Pregnancy Register by the CPRD and datasets made available to the project analyst. Weight and height data were selected from the *Additional Clinical Details file* in CPRD. BMI was generated following the method described in Bhaskaran et al. (2013) [31]. A detailed examination of LARC events rates e.g. trends by age group, country, LARC type is reported elsewhere [28].

To determine whether the LARC removal was for the purpose of conception, events following a removal were examined to either confirm or refute that a pregnancy was planned (pre-defined Read code list, see Additional File 2). Refuting a planned pregnancy included codes for alternative prescribed contraception, the menopause, or an unplanned pregnancy. Confirmation of a planned pregnancy included codes indicating pregnancy attempts or a planned pregnancy (e.g. a prescription of folic acid (see Channon et al. [28] for full details)). For the LARC removal to be considered associated with a pregnancy-planning event, the study team determined that the pregnancy should be within 456 days of the LARC event (based on an estimation of maximum 12-month conception period and 12 weeks to confirmatory scan, see full list of rules/assumptions in Additional File 3). The quality of BMI recording in the CPRD was explored. Only BMIs that were recorded within three years of either a pregnancy start date or the last LARC event for those with no associated pregnancy were included. Since the analysis for this study is descriptive in nature, appropriate statistics including frequency, percentage, mean, standard deviation, median and interquartile range were used to describe the characteristics of the data. No formal regression analysis was performed. Data analysis was done in SPSS version 25.

WP2 consisted of three components:

i) Understanding the policy context: UK policy and clinical guidelines pertaining to use (and in particular, removal) of LARCs were reviewed and collated.
ii) Understanding stakeholders experiences and views: qualitative surveys, using closed and open text questions, were designed for the different groups of stakeholders to develop an understanding of typical preconception pathways related to LARC/LARC removal and the inter-relationship between discussions about overweight/obesity and family planning from the perspectives of LARC users and service providers (See Table 1 for topics in each survey). The surveys were developed by the research team with feedback from practitioners and service users via the study management group.
iii) Stakeholder Advisory Groups (SAGs); Two SAGs, a LARC user SAG and a healthcare practitioner SAG, were created with participants recruited via the surveys. The role of the SAGs was to work with the research team to refine potential preconception WLI design options based on the surveys and policy and guideline findings.

**Table 1:** Survey topics for the stakeholder groups

LARC users	LARC removal HCPs
Experiences of discussing weight with HCPs Discussions of weight as part of contraceptive care Their knowledge of the risks of overweight/obesity in pregnancy Barriers and facilitators to the introduction of a WLI involving delaying LARC removal at LARC removal appointments	Experiences of discussing weight with patients Preconception care provision The discussion of weight in context of preconception health Barriers and facilitators to the introduction of a WLI involving delaying LARC removal at LARC removal appointments Views on acceptability of, and whether recruiting to such a WLI as part of their practice would be practicable

Survey responses to closed or multiple-choice questions were described descriptively. A computer assisted qualitative data analysis software package (NVivo 12) was used to manage open-text qualitative survey data. Data were analysed thematically and coded inductively. Inductive thematic analysis was considered appropriate since this approach is known to facilitate the exploration of similarities and differences across large datasets [32], allowing for variation in responses within and between LARC users and HCPs to be identified and explored. The six phases of thematic analysis proposed by Braun and Clarke [32] were used to guide the analytic process, the results of which were discussed at regular team meetings. The LARC user and HCP datasets were coded separately during the first stage of analysis with 20% of the surveys double-coded to ensure reliability. Once each dataset had been fully coded, a copy of each was merged. The resulting coding framework and dataset was considered as a whole and analysed by two members of the study team in order to identify key and cross-cutting themes, and to highlight areas of similarity and areas of difference across the LARC user and HCP groups. The SAGs were recorded, transcribed and the main findings summarised descriptively.

## Results

The findings of the two work packages are described separately: Work Package 1 results use routine data to identify the numbers of eligible women that could be potentially recruited into to a preconception WLI study. To do this the results are divided into four sections: (A) LARC use; (B) pregnancy within 1 year and 3 months of LARC removal; (C) codes to refute or confirm that the pregnancy was planned; (D) BMI status. Work Package 2 results describe the contextual factors firstly of the relevant policy and guidelines, secondly the survey findings reflecting the views of the LARC users and practitioners and finally the reflections on these findings from the SAGs.

### Work Package 1: The use of routine data to identify women using LARCs and planning a pregnancy

A total of 2,632,871 records from women of reproductive age (16 to 48 years old) were identified from the CPRD between 1^st^ January 2009 to 31^st^ December 2018, of whom 318,040 (12.1%) women were in the pre-defined study population (at least one consultation identified as LARC-related) (see Additional file 4 for the generation and exclusions of the study population).

#### LARC-related consultations

A total of 315,755 (12.0%) women in the study population had at least one LARC event (total of 929,099 LARC events). The proportion of women with at least one LARC event decreased over the time period from 2.2% in 2009 (approx. 42 LARC events per practice) to 1.3% in 2018 (approx. 24 LARC events per practice). Table 2 shows LARC-related consultations broken down by consultation type. Individuals in our study population could experience more than one code over the study period. A total of 108,987 women had a LARC removal (34.5% of the study population); 12,615 (12%) had more than one LARC removal code recorded over the study period.

**Table 2:** LARC events by consultation type

	**Unique number of events**	**Number of patients with at least one event** ***N* = 315,755**
**LARC consultation type†**		
Insertion	592,402	269,999 (85.5%)
In situ	222,555	127,909 (40.5%)
Removal	123,204	108,987 (34.5%)
Total number of events	938,161**†**	

^†^ *N* = 9,062 codes were double counted due to three codes (IUD – Not otherwise specified (NOS), Subcutaneous contraceptive NOS, and Subcutaneous contraceptive) being coded as both LARC in situ and LARC insertion

#### Pregnancy within 1 year and 3 months of LARC removal

A total of 16,394 pregnancy events occurred in the study period among 15,297 (4.8%) women in the study population (*n* = 315,755) (see Additional file 4). 4,753 (29.0%) pregnancies did not have a LARC removal code before pregnancy started (presumably, in most cases, due to removal by other services) and 299 (1.8%) had a removal code between pregnancy start and end (possibly due to incorrect removal date). For the remaining 11,342 (69.2%) pregnancies, the median duration from LARC removal to conception was 109 days (25^th^ to 75^th^ centiles = 47 to 220 days).

#### Codes to refute or confirm that the pregnancy was planned

To determine whether the LARC removal was for the purpose of conception, events between the two were examined to either confirm or refute that the pregnancy was planned (Additional file 5). 474,109 different scenarios/pathways to a pregnancy event or not, within 315,755 patients were observed. The majority (77%) were probably not planning a pregnancy, with only 1.7% planning and 2.8% possible planning a pregnancy.

#### BMI status

Of all the 474,044 scenarios included, 292,803 (62%) had a valid BMI measurement in the same patient record within three years of the LARC event (constant across all BMI groups (range 60–67%)) (Additional file 5). In those planning a pregnancy, 54% had a BMI ≥ 25 and 28% had BMI ≥ 30 (Table 3). If it is assumed that women with no recorded BMI had a healthy BMI, the proportion of women either overweight or obese, planning a pregnancy would decrease to 35.6% (2,314/6,506) and 18.4% (1,198/6,506) respectively.

**Table 3:** Completeness of BMI and BMI categories – all n(%)

Study Classification	N women	BMI not recorded	BMI recorded*	Overweight and Obese (BMI 25 +)	Obese (BMI 30 +)	Morbidly obese (BMI 40 +)
Planning a pregnancy†	6,506 (1.4)	2,178 (33.5)	4,328 (66.5)	2,314 (53.5)	1,198 (27.7)	204 (4.7)
Planning a pregnancy††				35.6%	18.4%	3.1%
Possibly planning a pregnancy†	10,902 (2.3)	3,868 (35.5)	7,034 (64.5)	3,554 (50.5)	1,666 (23.7)	190 (2.7)
Possibly planning a pregnancy††				32.6%	15.3%	1.7%
Probably not planning a pregnancy†	383,346 (80.9)	146,191 (38.1)	237,155 (61.9)	124,683 (52.6)	60,536 (25.5)	9,537 (4.0)
Probably not planning a pregnancy††				32.5%	15.8%	2.5%
Not enough information†	73,290 (15.5)	29,004 (29.6)	44,286 (60.4)	23,657 (53.4)	11,751 (26.5)	1,897 (4.3)
Not enough information††				32.3%	16.0%	2.6%
**TOTAL**	**474,044**	**181,241 (38.2)**	**292,803 (61.8)**			

^*^within 3 years of LARC event

^*†*^*denominator* = *women with a BMI recorded*

^*††*^* denominator* = *total women; Excluded 65 women who have unclear coding*

### Work Package 2: Understanding context

#### UK Policy and guidelines

A total of 15 Policy documents were identified as relevant, including publications from Faculty of Sexual and Reproductive Healthcare (FSRH), National Institute for Health and Care Excellence (NICE), British Pregnancy Advisory Service (BPAS) and Royal College of Obstetricians and Gynaecologists (RCOG) [33–47]. Although several documents included reference to LARC use and weight these focussed on issues of safety and confirming the lack of evidence of an association between LARC use and weight gain [41, 46, 47]. Optimizing preconception weight was identified as important [33–35, 38, 39] and preconception care and contraception-related encounters were identified as opportunities to discuss this, but there is little guidance provided on how such discussions would take place.

#### Surveys with LARC users and HCPs

The online LARC user survey was completed by 243 people. The majority of respondents (85.2%) self-classified as currently overweight, 41.6% of LARC users were either currently pregnant or had been previously and 58.4% of LARC users were planning to conceive in the future (Table 4).

**Table 4:** LARC user characteristics

	Currently n/%	In the past n/%	Planning in the future n/%
Use of LARC	128/52.7%	115/47.3%	N/A
Overweight	207/85.2%	36/14.8%	N/A
Pregnant	12/4.9%	89/36.6%	142/58.4

The practitioner survey was completed by 100 HCPs, mainly doctors (79%), across primary care (46%) and sexual health clinics (43%) settings. Most HCPs had been in their role for more than 6 years (92%) and were involved in both LARC insertion and removal (94%).

The survey results reported here focus on the key question of acceptability and practicability of delaying LARC removal in order for people to take part in a preconception weight loss programme. The findings describing experiences of weight related discussions in other health appointments are also included as important contextual information.

### General health consultations including weight as part of the discussion

Many HCPs considered the provision of healthy lifestyle advice part of their role, with discussions about weight being characterised as no different to discussions on other topics such as smoking; consequently some described introducing weight opportunistically, at any healthcare appointment.

“It needs discussing & we have a responsibility as health professionals not to shy away from this conversation” (HCP23)

Others depicted their approach as more selective, describing for instance how they would only discuss weight if it was specifically relevant to the appointment at hand, and with an awareness that such conversations could be difficult.

For many LARC users there was a sense that, whether they wanted it or not, discussion of weight was an inevitable part of health consultations in a variety of contexts, including circumstances when it did not seem particularly relevant:

*“Almost every medical professional I have ever seen has suggested symptoms of everything from a chest infection to a sprained wrist would be improved if I lost weight.”(LARC User (LU)4)*

This generalised approach seemed to be particularly the case for LARC users with higher BMIs: the proportion of LARC users reporting that weight had been discussed at general appointments was greater in women who self-reported that their BMI ≥ 30, compared to those with BMI < 30 (61.0% compared to 32.6% respectively).

Very few LARC users reported being provided with practical advice or support, and when advice was provided it was often perceived as obvious and unhelpful: *“I was told I was overweight and to lose it. There was no help offered at all (LU193)”.* A few examples of positive experiences were reported however; LARC users described how HCPs had been supportive, non-judgemental and had offered practical, long-term help.

### Preconception consultations and discussion of weight

Preconception consultations were reported to be rare, and although weight was seen by most practitioners as relevant, it was not considered a priority given the range of other topics to be covered (e.g. folic acid, rubella status and smoking).

LARC users reported that they had received recommendations from healthcare professionals to lose weight in order to increase likelihood of conception (and access IVF) during preconception-related consultations, but with no practical advice or support.

“I had been trying (to conceive) for over a year and told I was too fat and to lose weight so it would magically happen.”(LU23)

### Acceptability of discussing weight during a LARC removal and delaying LARC removal

Many LARC users (46.8%) described feeling uncomfortable at the prospect of discussing weight at LARC removal appointments, in contrast to the majority of HCPs (65%) who would feel comfortable introducing the subject of weight with patients attending for LARC removal. In answer to the specific question about whether it would be acceptable to ask LARC users who have a BMI ≥ 25 to delay LARC removal in order to attend a WLI prior to trying to conceive, 39.9% of LARC users and 63.6% of HCPs stated that they felt it would be acceptable. It was unacceptable to 29.6% of LARC users and 17.2% of practitioners, with the remaining 30.5% of LARC users and 19.2% of HCPs being unsure.

When asked to expand on reasons for their answer, practitioners and LARC users identified facilitators and barriers to this proposal. Since many themes from the two stakeholder groups reflected similar core issues, their experiences are also reported here within the five broad themes (with their perspectives highlighted).

i) Sensitivity of the topic
ii) LARC user experiences, knowledge and beliefs
iii) Healthcare Practitioner role and skills
iv) Setting
v) Ethical Implications

#### The sensitivity of weight as a topic area

Both LARC users and HCPs considered conversations regarding weight at LARC removal appointments difficult because of the sensitivity of the topic, echoing themes from the discussion of weight in general consultations. LARC users described feeling ashamed, attacked and judged during health consultations for having a raised BMI and practitioners were aware of the potential to upset patients, with possible repercussions for them and their relationship.

“I know I am overweight, I feel helpless when it's pointed out.”(LU108)

“Following a recent complaint when I discussed weight with a patient at a contraceptive review, it has made me wary about initiating discussions.”(HCP64)

There was however some ambivalence within both groups, with participants both acknowledging discomfort and retaining the belief that weight-focussed conversations were needed. Some LARC users appreciated HCPs’ good intentions and recognised the importance of such conversations, and similarly HCPs described the necessity of discussing weight as a health risk with patients despite potential negative outcomes.

“Because weight is a very personal thing and it sometimes feels like an attack even though with a health professional I know it is in my best interest”(LU44)

“I am uncomfortable about it because it upsets women but I think it is my medical duty to mention it so try and do it as tactfully as possible.”(HCP87)

#### LARC user experiences, knowledge and beliefs

The potential acceptability of conversations relating to weight was described by both stakeholder groups as being very individual and dependent on the woman’s personal circumstances, such as age, history (e.g. in relation to weight, fertility, previous pregnancies, mental health, previous experience in consultations), current weight status and their attitudes towards this.

One of the main facilitators of this potential preconception WLI for LARC users was their awareness of the impact of weight on health, including the risks of overweight in pregnancy. Some reflected that communication of the risks of obesity in pregnancy was essential to allow patients to make an informed choice, to maintain autonomy and that this discussion could be a motivator to engage in a WLI.

One of the identified barriers to introducing a preconception WLI was LARC users’ repeated experiences of having a practitioner simply mention weight without offering support; an approach which is for them, at best, ineffectual. Many practitioners were aware that service users were “*fed up with health care professionals telling them they are overweight when they already know they are overweight” (HCP27).* Whilst policy documents highlight evidence which shows that LARCs do not lead to weight gain, some LARC users maintained a belief that they do, and as such being advised to keep a LARC in place in order to lose weight may not make intuitive sense. Many LARC users also mistrusted the emphasis on BMI, not believing that weight maps onto health as closely as suggested by HCPs. In turn, this belief led to further questioning of assertions about risk, and the desire for clearer information/statistics to support informed choice.

*“I know all about the risks but lots are correlation rather than causation. But only 34 weeks so still a chance to have a double chance of still birth and increased risk of SIDS. Don't worry about the confounding socio-economic factors behind those statistics I am fat so I am doomed.” (LU6)*

#### HCPs’ skills

Discussing health issues such as weight was identified by both stakeholder groups as an integral part of a medical professional’s role, and having that discussion in the context of an ongoing relationship was crucial for success. LARC users stressed the importance of the HCPs communication skills (eg sensitive, non-judgmental, putting them at ease etc.). Having adequate time, not treating the conversations like “tickbox” exercises, sensitivity towards terminology likely to cause offense (such as ‘obese’ or ‘fat’), and having an understanding of difficulties associated with losing weight were all seen as important facilitators to effective communication.

Lack of skill in this area of practice was a concern for both groups: many LARC users suggested that HCPs lacked the required skills to deal with the sensitive topic of weight management. Similarly, HCPs acknowledged they could feel uncomfortable discussing weight with patients, particularly when providing preconception advice, and explained that they would benefit from training and guidance to ensure they were taking the right approach. HCPs wanted accurate information regarding risks of raised BMI in pregnancy, and/or effective weight loss advice, to facilitate successful discussions with patients.

“*Making us feel crap about our choices and our bodies, is not a way to motivate anyone.” (LU8)*

*“Staff will feel uncomfortable unless they have some guidance on how and when to phrase things.” (HCP87)*

#### Context of the consultation and service setting

LARC users thought preconception and pregnancy could be considered windows of opportunity for introducing weight loss conversations, to maximise fertility and improve health in pregnancy. Potential benefits to the unborn child were recognised as a facilitator to fostering healthy behaviours.

*“Because the healthier you are the better chance you have of getting pregnant and having a problem free pregnancy. Wouldn’t any mother want that?” (LU150)*

The reason for patients requesting LARC removal was thought to be relevant to whether weight should be introduced. Participants suggested that if patients requested LARC removal for reasons other than wanting to conceive (e.g. side effects such as heavy periods, weight gain, changes in mood) then it may not be appropriate to raise the topic of weight.

One key barrier identified by both stakeholder groups was that weight is not seen as relevant to contraception, and that discussion of weight or health at contraception appointments is not appropriate, unless explicitly raised by the service user.

*“I'm not there presenting with a health complaint, so why discuss weight?” (LU5)*

Both groups also raised concerns about the logistics of asking women to delay LARC removal during contraceptive appointments, given that patients’ have “*to wait months for an appointment to remove my implant”* and brief appointments.

*“It would be really hard to have this kind of conversation in a very short appointment in a successful and productive way..”(LU44)*

*“In my surgery, patients book in without prior counselling for a 10 min (coil) or 20 min (implant) removal so time is limited. We would have to change our system.”(HCP93)*

Finally, LARC users described feeling vulnerable talking about birth control, especially in the context of intimate examinations in LARC removal appointments, and felt it would be inappropriate to instigate discussions regarding weight.

*“For some females it can be incredibly nerve wrecking going to have a discussion with a total stranger about birth control. Especially if it’s the coil where they are at the most intimate part of a female’s body then they start talking about the persons weight!” (LU75)*

#### Ethical implications of requesting LARC removal delay

The largest group of responses identifying barriers to the idea of delaying LARC removal in order to lose weight expressed what we have described as ethical barriers which relate to personal choice, conception decision-making, impact on the care pathway and the exclusion of non-LARC users.

### Individual freedom to choose

LARC users and HCPs highlighted the ethical implications of what could be interpreted as an imposition of a contraceptive decision on patients. Some LARC users referred to discrimination against people with raised BMI and the perceived insinuation that

*“fat people shouldn’t breed”. (LU4)*

They felt that state involvement in personal reproductive decisions was unacceptable and they were concerned that people may feel pressured into agreeing to delay LARC removal, acknowledging that HCPs, despite acting with best intentions, may be unaware of this consequence. However, HCPs also acknowledged the potential to pressure patients in a “doctor-led approach”.

*“I would find it ethically complex to suggest to women that their freedom of choice to have their contraception removed may be compromised by their weight..” (HCP57)*

*“…this is already happening in places, with women reporting having been 'refused' to have their implant/coil removed, conditional on losing weight. That may not be what the healthcare professional thought they were saying, but that is the message women are hearing.” (LU8)*

*“Because you’re denying someone the right to get pregnant due to their weight. That is a breach of their human rights.” (LU180)*

### Complexity of timing in conception decisions

The timing of decision making about conception was characterised as complex: LARC users identified multiple influential factors including relationships, studies, career choices, sibling age-gap, and financial implications. LARC users and HCPs commented on the risk of delay for older patients or those with fertility issues.

*“How dare anyone think they have the right to intervene in such a monumentally personal decision for a couple?” (LU8)*

*“Personally, I have a coil and have planned it’s duration around my university studies and finances. I can’t imagine I am the only one. I have planned when I will begin a family regardless of my weight or what a doctor tells me.” (LU109)*

*“…may not achieve anything other than delaying pregnancy and LARC removal—so you end up with older overweight people getting pregnant.”(HCP42)*

### Impact on care pathway

Participants suggested that if patients were aware they would be asked to delay LARC removal due to weight, this could discourage them from having a LARC fitted, or may lead them to go elsewhere for removal. LARC users questioned what the process would be if patients declined to attend a WLI, or if they were unsuccessful at losing weight, and whether this would impact on their care or whether they were “allowed” to have a baby.

### Exclusive intervention

Finally, participants highlighted how people may feel excluded from an intervention targeting women trying to conceive, suggesting that an effective weight-management intervention should be offered on a population level.

*“As a side point, I'd like to be a healthy weight for me and the fact that I can't get help with my weight or energy levels because I'm not or wasn't trying to conceive instils a bitter sort of anger” (LU161)*

### Work Package 2: Stakeholder Advisory Groups (SAGs)

Three LARC users attended the virtual LARC user SAG meeting and 34 HCPs attended the healthcare practitioner SAG. Feedback confirmed and consolidated the main themes from the survey, providing particular clarity and input in three areas:

#### Acceptability of asking women to delay LARC removal

The mechanism of delaying LARC removal as an approach to recruiting to a preconception intervention would be too blunt an approach: However, with nearly 40% of women thinking it could be acceptable, and with over 60% of practitioners willing to consider it, if managed well and in the right circumstances, it could be one route into a preconception WLI and could be incorporated into further discussions of WLI design.

#### Components of a preconception WLI

Beyond the idea of having a positive focus, which both stakeholder groups agreed on, participants held mixed views on WLI key components. Disagreement remained in relation to whether there should be a specified weight loss goal, what the BMI threshold for inclusion should be, and whether use of meal replacements would be appropriate. These issues were identified as areas to take forward into further stakeholder work.

#### Exploring interviewees experiences

LARC users suggested that further stakeholder work via individual interviews should address questions that enable the study team to build on positive experiences, exploring what has been helpful weight-wise for interviewees in the past, and including examples of positive conversations about weight, whilst also exploring unacceptable language in more depth. Practitioners thought interviews should explore practitioners’ need for training, what aspects of their previous experience might assist successful conversations about weight, and the impact of practitioners’ own weight.

## Discussion

This mixed-methods study was designed to explore the potential opportunity for a preconception weight loss intervention being offered to those requesting LARC removal as part of their preparation for conception. Overall, the quality and completeness of the routine datasets would not be adequate to identify potential participants for such an intervention, without additional input from services. Service users and healthcare providers understood the importance of preconception health: However, they identified many concerns about discussion of weight in this context and barriers associated with the proposed intervention, including service-user past experiences of weight related interventions, practitioner skills, the setting and ethical implications of the proposed intervention.

The findings of the study are considered in terms of the study objectives and then in the wider context of preconception weight loss interventions.

Objectives 1 and 2: The routine datasets relating to LARC use and removal were complex: The lack of connection between the datasets of different parts of the infrastructure, i.e. sexual health services and primary care, combined with incomplete and often imprecise data, means it would not be possible to reliably identify women who request a LARC removal with an intention to become pregnant simply through NHS records. Similarly, although there is a BMI recorded within three years of a LARC related consultation for an average of 62% of women, the quality of the routine data would make it an unreliable route to take to identify women who could be eligible for the proposed weight loss intervention. Therefore identification of women by healthcare professionals in primary care and at sexual health services at LARC removal appointments would potentially provide the most effective way to identify eligible women.

Objective 3: Practitioners were generally willing to discuss weight in consultations with eligible women and some felt it was an integral part of their role. The majority would also consider inviting women to take part in a preconception weight loss study incorporating a delay in LARC removal. However, the practitioners raised multiple barriers to both tasks including practical concerns such as time available in consultations, their communication skills in relation to weight, the sensitivity of the topic of weight and, specifically in relation to recruiting to the weight loss intervention, the appropriateness of having such a discussion at a LARC removal. Any preconception weight loss intervention recruiting via practitioners in sexual health services and primary care would need to overcome these barriers in order to operate successfully.

Objective 4: There was a wide range of views from women on the acceptability of delaying LARC removal to take part in a preconception weight loss intervention. The key factors that could potentially make it acceptable would be sensitive, person-focussed communication that acknowledges and works with the patient’s prior experience of weight difficulties and puts the service user in control of the decision-making. However, from their descriptions of their lived experience, the logistics of such an intervention in an overstretched service and the quality of the communications about weight, means these conditions would be unlikely to be met. Timing of a discussion about weight in relation to pregnancy is crucial: The decision to have a baby is often complex, thought through (with partner), and part of an emotional, social and financial web of circumstances. Whilst many have LARC removal without it being part of a decision to get pregnant straight away (e.g. because of side effects), they would still not want to delay removal. For those where it is a deliberate step to pregnancy the removal appointment is considered too late in the decision-making process. Alongside these practical and personal considerations, there were also overarching concerns about the fundamental message of such an intervention and that the ethos of the intervention undermines the woman’s right to choose when she could conceive. Whilst there was never any intention that the intervention would remove the woman’s choices, some stakeholders identified a discriminatory underlying message in the proposed intervention design. However, the median time from LARC removal to pregnancy of 109 days, as identified in the routinely collected data, would present an opportunity for many women to initiate a less-intensive weight management intervention, that may continue in pregnancy and have a positive impact on pre-pregnancy weight and gestational weight gain.

On balance, in its basic form, an intervention comprised of delaying LARC removal in order to take part in a weight loss programme prior to conception, faced significant barriers in the context of current service delivery. However, including this as one option in a preconception health and weight loss programme, that was designed with the key tenet of informed choice at its heart, could be acceptable and potentially feasible.

Weight management and weight in relation to health are very complex contexts: Individuals’ beliefs about the health impact of weight and how it fits with other health issues is critical to their engagement with any weight intervention and this is true for both the service user and the practitioner. The lack of BMI in the data is a limitation in this study but it is also a very telling gap in the dataset: With only 62% of people having a BMI recorded, it is clearly not a measure that HCPs are taking routinely. This makes it harder to introduce weight into the consultation opportunistically but may also reflect the ambivalence practitioners have about raising weight or their beliefs about its relative importance in health. It could be assumed that those with higher BMI are more likely to have been weighed, but this selective approach risks reinforcing the practice that distresses service users, of practitioners assessing their weight “by eye”, which is experienced as very judgemental.

The nuanced relationship between weight and health came through the stakeholder work and may be particularly relevant to preconception and pregnancy. It is a time when there are multiple health messages around diet, exercise, smoking, alcohol, folic acid etc. so it may be unhelpful to single out weight. An overly weight-centred approach could potentially exacerbate the experience of weight stigma that our service users described so eloquently in their survey responses. Many gave examples of being told to lose weight without being offered any support or guidance, an experience which would inform their response to this type of intervention. However, if weight management was incorporated into a wider narrative around healthy eating, as part of a lifestyle-focussed preconception intervention, it may resonate more strongly with their wish to create a healthy environment for their baby. Also, if it included guidance from appropriate professionals such as dieticians and kinesiologists, the offer may be more acceptable. These possibilities need to be explored in more depth, as does the view, expressed in both groups of stakeholders, that contraception is not part of wider health, that somehow it is a separate issue. In order to improve preconception health, programmes need to reflect these complexities and there needs to be greater awareness of the importance of weight and preconception health at a population level instead of stigmatisation of pregnant women.

The main strengths of this study have been the use of routine data to consider viability and the engagement with key stakeholders in exploring the acceptability and practicability of the idea without using resources for a feasibility pilot study. There are limitations in using CPRD as not all GP practices are included and sexual health clinic data are not linked: In any future trial using routine data these issues would need to be resolved. There were also limitations in the extent and reach of recruitment; despite our best efforts at advertising and use of social media the response rate from LARC users was not as high as we hoped, maybe because the specificity of our target group made it difficult to convey the study succinctly. Recruiting through professional events was successful in many ways as it did not cut across practitioners’ clinical time, but it did result in a group of contributors with many years experience and also with fewer nurses than would be representative of the practitioner group.

Future research is needed to explore ways to overcome the barriers experienced by healthcare staff in discussing weight as part of preconception care. Very often the focus falls on pragmatic barriers such as time in consultations, but this study has underlined the potential importance of topics such as professionals' beliefs about the impact of weight on health, their professional remit in relation to weight and the links between contraception services and general health. We know from other studies in the general population, e.g. the BWel study, that a brief opportunistic intervention by a primary care practitioner introducing the topic of weight can be acceptable to patients [48]. Developing ways to support practitioners to sensitively and effectively raise the issue of pre-conception weight management needs to be a priority as unless these barriers are reduced or removed and the quality of the communication is improved, a population-based preconception weight loss intervention based in the NHS will not be viable. The individual’s experience of preconception interventions also seems to be a crucial missing piece in the evidence as it currently stands. With often very low recruitment and retention rates, even in the context of fertility treatment where motivation would be high, there is a pressing need to have a clearer understanding of the lived experience. The second phase of this study explores some of these issues via stakeholder interviews: The ongoing trials of preconception interventions will hopefully include process evaluations to improve our understanding of the barriers and facilitators of engagement for both practitioners and service users which will move this field forward.

## Conclusions

At the present time it would not be viable to develop an intervention that asks LARC users with a raised BMI to delay removal of LARC to participate in a targeted preconception weight loss intervention, due to significant barriers described by women and healthcare professionals. A broader population-based preconception programme including discussions about weight at contraception-related appointments could be acceptable. However, in order for a preconception weight loss programme to succeed it would need to overcome current barriers, including the knowledge in the general population about the benefits of preconception health and the provision of guidance and training to healthcare providers in communication about weight.

## Supplementary Information

**Additional file 1. **Defining LARC events using (a) read codes and (b) BNF prescription codes.

**Additional file 2. **Clinical codes.

**Additional file 3. **Assumptions agreed by the Plan-it study team in advance.

**Additional file 4. **Participant flow diagram.

**Additional file 5. **Defining the groups based on LARC use, events related to planning a pregnancy andcontrary events, and conception.

## Data Availability

The data that support the findings of this study are available on reasonable request from the corresponding author (SC). The data are not publicly available as they contain information that could compromise research participant privacy/consent.
