# Supplementary material for: The acceptability of asking women to delay removal of a long-acting reversible contraceptive to take part in a preconception weight loss programme: a mixed methods study using qualitative and routine data (Plan-it)
Source: BMC Pregnancy Childbirth. 2022 Oct 18;22:778. doi: 10.1186/s12884-022-05077-0 (PMC9580156; doi:10.1186/s12884-022-05077-0)
Supplement: Supplementary file 1 — Additional file 1. Defining LARC events using (a) read codes and (b) BNF prescription codes. [file 12884_2022_5077_MOESM1_ESM.docx]

# Additional File 1: Defining LARC events using (a) read codes and (b) BNF prescription codes.

Inclusion Criteria: Women of reproductive age (16-48 years old) who have a LARC related event (insertion/in situ/removal) during 01 JAN 2009-31 DEC 2018.

#### (a) Read codes

| **MedCode** | **Label** | **LARC consultation type** | **Type of LARC** |
| --- | --- | --- | --- |
| 8354 | [V]Coil in situ | In situ | IU device |
| 10957 | [V]Coil check | In situ | IU device |
| 45339 | [V]Coil maintenance | In situ | IU device |
| 20658 | [V]Coil check | In situ | IU device |
| 107776 | Retained intrauterine contraceptive device | In situ | IU device |
| 12029 | [V]Intrauterine contraceptive device present | In situ | IU device |
| 6306 | [V]Intrauterine contraceptive device present | In situ | IU device |
| 2145 | [V]Intrauterine contraceptive device present | In situ | IU device |
| 47908 | Intrauterine contraceptive device 6 week check | In situ | IU device |
| 22914 | Intrauterine contraceptive device annual review | In situ | IU device |
| 6265 | [V]Intrauterine contraceptive device check | In situ | IU device |
| 20557 | [V]Intrauterine contraceptive device check | In situ | IU device |
| 21515 | [V]Intrauterine contraceptive device check | In situ | IU device |
| 5917 | IUD checked - no problems | In situ | IU device |
| 225 | IUD in situ | In situ | IU device |
| 20392 | Coil follow-up administration | In situ | IU device |
| 107433 | Uterine perforation by intrauterine contraceptive device | In situ | IU device |
| 54183 | [V]Surveillance of (intrauterine) contraceptive device | In situ | IU device |
| 51242 | IUD follow-up admin. NOS | In situ | IU device |
| 21421 | IUD follow-up administration | In situ | IU device |
| 63924 | FP1002 due next with new IUD | In situ | IU device |
| 44917 | IUD check - 2nd call | In situ | IU device |
| 40783 | IUD check - 1st call | In situ | IU device |
| 45820 | IUD check - 3rd call | In situ | IU device |
| 13004 | IUD change due | In situ | IU device |
| 37020 | IUD - defaulted from check | In situ | IU device |
| 19950 | IUD check due | In situ | IU device |
| 445 | IUD check | In situ | IU device |
| 20538 | Mechanical complication of coil | In situ | IU device |
| 112311 | Infection associated with intrauterine contraceptive device | In situ | IU device |
| 29106 | Mechanical complication intrauterine contracep.device (IUCD) | In situ | IU device |
| 23439 | Bleeding due to intrauterine contraceptive device | In situ | IU device |
| 17735 | Mechanical complication of intrauterine contraceptive device | In situ | IU device |
| 104471 | Intrauterine contraceptive device threads seen | In situ | IU device |
| 108636 | Bleeding due to intrauterine contraceptive device | In situ | IU device |
| 30765 | IUD partially expelled | In situ | IU device |
| 7888 | IUD checked - problems | In situ | IU device |
| 5564 | IUD threads lost | In situ | IU device |
| 52230 | Intrauterine contraceptive device annual review by telephone | In situ | IU device |
| 6050 | Change of intrauterine contraceptive device | In situ | IU device |
| 88224 | Intrauterine contraceptive device fit by another GP practice | In situ | IU device |
| 22652 | Replacement of intrauterine contraceptive device | In situ | IU device |
| 95906 | Intrauterine contracep device checked by other hlth provider | In situ | IU device |
| 2738 | IUD in situ from other agency | In situ | IU device |
| 93404 | Subcutaneous contraceptive in situ | In situ | Implant |
| 26477 | Check of subcutaneous contraceptive | In situ | Implant |
| 96963 | Subcutaneous contraceptive implant palpable | In situ | Implant |
| 106241 | Contraceptive implant removal invitation | In situ | Implant |
| 98121 | Mirena coil check | In situ | IU system |
| 21114 | Coil intrauterine contraceptive device procedure | Insertion | IU device |
| 10614 | Coil contraception | Insertion | IU device |
| 24497 | [V]Reinsertion of coil | Insertion | IU device |
| 6064 | [V]Coil insertion | insertion | IU device |
| 21365 | [V]Reinsertion of coil | Insertion | IU device |
| 17573 | IUD contraception | Insertion | IU device |
| 10754 | [V]Reinsertion of intrauterine contraceptive device | Insertion | IU device |
| 9226 | Fitting of intrauterine contraceptive device | Insertion | IU device |
| 6772 | Intrauterine contraceptive device procedure | Insertion | IU device |
| 4401 | [V]Reinsertion of intrauterine contraceptive device | Insertion | IU device |
| 31602 | [V]Reinsertion of intrauterine contraceptive device | Insertion | IU device |
| 6941 | Introduction of intrauterine contraceptive device | Insertion | IU device |
| 2144 | [V]Intrauterine contraceptive device insertion | Insertion | IU device |
| 3882 | [V]Intrauterine contraceptive device insertion | Insertion | IU device |
| 26317 | Intrauterine contraceptive device procedure NOS | Insertion | IU device |
| 17980 | [V]Intrauterine contraceptive device insertion | Insertion | IU device |
| 53523 | Other specified intrauterine contraceptive device | Insertion | IU device |
| 182 | IUD fitted | Insertion | IU device |
| 42310 | Post-coital IUD fitted | Insertion | IU device |
| 38544 | 'Morning after' IUD fitted | Insertion | IU device |
| 485 | IUD re-fitted | Insertion | IU device |
| 17440 | Intrauterine device procedure | Insertion | IU device |
| 40402 | Coil contraceptive claim | Insertion | IU device |
| 99571 | IUD contraceptive claim | Insertion | IU device |
| 27859 | IUD contraceptive claim | Insertion | IU device |
| 27872 | IUCD contraceptive claim | Insertion | IU device |
| 55297 | FP1002 - IUD insertion claim | insertion | IU device |
| 103642 | Insertion of etonogestrel radiopaque contraceptive implant | Insertion | Implant |
| 104317 | Insertion of subcutaneous contraceptive claim | Insertion | Implant |
| 22950 | Insertion of subcutaneous contraceptive | Insertion | Implant |
| 101819 | Reinsertion of subcutaneous contraceptive | Insertion | Implant |
| 70535 | Insertion of hormone into subcutaneous tissue | Insertion | Implant |
| 90157 | Replacement of hormone in subcutaneous tissue | Insertion | Implant |
| 17183 | Insertion of hormone implant | Insertion | Implant |
| 103004 | Subdermal etonogestrel implant insertion ESA | Insertion | Implant |
| 100958 | Insert subcutaneous contraceptive implnt othr healthcre prov | insertion | Implant |
| 7255 | Introduction of Mirena coil | Insertion | IU system |
| 102368 | Intrauterine system contraception | Insertion | IU system |
| 2795 | IUD - NOS | Insertion/In situ | IU device |
| 22951 | Subcutaneous contraceptive NOS | Insertion/In situ | Implant |
| 26092 | Subcutaneous contraceptive | Insertion/In situ | Implant |
| 20424 | [V]Removal of coil | Removal | IU device |
| 18341 | [V]Removal of coil | Removal | IU device |
| 7379 | Removal of intrauterine contraceptive device NEC | Removal | IU device |
| 11561 | [V]Removal of intrauterine contraceptive device | Removal | IU device |
| 5252 | [V]Removal of intrauterine contraceptive device | Removal | IU device |
| 27929 | [V]Removal of intrauterine contraceptive device | Removal | IU device |
| 446 | IUD removed | Removal | IU device |
| 106270 | Intrauterine contraceptive device removal invitation | Removal | IU device |
| 107556 | Expulsion of intrauterine contraceptive device | Removal | IU device |
| 26476 | IUD removal awaited | removal | IU device |
| 25680 | Removal of contraceptive coil from pouch of Douglas | Removal | IU device |
| 6225 | Removal intrauterine contracept device from pouch of Douglas | Removal | IU device |
| 32611 | Removal of displaced intrauterine contraceptive device | Removal | IU device |
| 22945 | IUD fallen out | Removal | IU device |
| 22946 | IUD expelled | Removal | IU device |
| 95476 | Intrauterine contracep device removed by other hlth provider | Removal | IU device |
| 107048 | Removal of subcut contraceptive implant using US guidance | Removal | implant |
| 26260 | Removal of subcutaneous contraceptive | Removal | implant |
| 104861 | Removal of subcutaneous contraceptive claim | Removal | implant |
| 22875 | Removal of hormone implant from subcutaneous tissue | Removal | implant |
| 71272 | Removal of hormone implant from subcutaneous tissue | Removal | implant |
| 103620 | Removal of etonogestrel radiopaque contraceptive implant | Removal | implant |
| 101010 | Remov subcutaneous contraceptive implant othr healthcre prov | Removal | implant |
| 18745 | Removal of Mirena coil | Removal | IU system |
| 109722 | Removal of intrauterine system | Removal | IU system |

####

#### (b) Prescription Codes

BNF chapters have been identified for inclusion (rather than specific prodcodes) from BNF

Chapters 7.3.2.2, 7.3.2.3 & 7.3.4.1.

| **prodcode** | **productname** | **bnf** | **bnfchapter** | **Type of LARC** |
| --- | --- | --- | --- | --- |
| 66331 | Ancora 375 Ag intrauterine contraceptive device (R.F. Medical Supplies Ltd) | 07030400/07030450 | Contraceptive Devices/ Intrauterine Contraceptive Devices | IU device |
| 66341 | Ancora 375 Cu intrauterine contraceptive device (R.F. Medical Supplies Ltd) |  |  | IU device |
| 1196 | Novagard type 6 Intrauterine device (Pharmacia Ltd) | 7030450 | Intrauterine Contraceptive Devices | IU device |
| 4904 | Gyne-T 380S intrauterine device (Janssen-Cilag Ltd) |  |  | IU device |
| 5265 | Multiload Cu375 intrauterine contraceptive device (Organon Laboratories Ltd) |  |  | IU device |
| 5405 | Nova-T 380 intrauterine contraceptive device (Bayer Plc) |  |  | IU device |
| 5647 | T-Safe 380A QL intrauterine contraceptive device (Williams Medical Supplies Ltd) |  |  | IU device |
| 12146 | Ortho-gyne type 4a iucd -t Intrauterine device (Janssen-Cilag Ltd) |  |  | IU device |
| 13678 | Ortho-gyne type 4b Intrauterine device T380S (Janssen-Cilag Ltd) |  |  | IU device |
| 14038 | Flexi-T 300 intrauterine contraceptive device (Durbin Plc) |  |  | IU device |
| 14295 | Flexi-T+ 380 intrauterine contraceptive device (Durbin Plc) |  |  | IU device |
| 14959 | TT380 Slimline intrauterine contraceptive device (Durbin Plc) |  |  | IU device |
| 18826 | Multi-Safe 375 intrauterine contraceptive device (Williams Medical Supplies Ltd) |  |  | IU device |
| 19143 | GyneFix intrauterine contraceptive device (Williams Medical Supplies Ltd) |  |  | IU device |
| 26488 | Neo-Safe T380 intrauterine contraceptive device (Williams Medical Supplies Ltd) |  |  | IU device |
| 33367 | Load 375 intrauterine contraceptive device (Durbin Plc) |  |  | IU device |
| 36984 | Mini TT380 Slimline intrauterine contraceptive device (Durbin Plc) |  |  | IU device |
| 36995 | UT380 Short intrauterine contraceptive device (Durbin Plc) |  |  | IU device |
| 38015 | UT380 Standard intrauterine contraceptive device (Durbin Plc) |  |  | IU device |
| 43404 | Steriload intrauterine contraceptive device (Farla Medical Ltd) |  |  | IU device |
| 45739 | Optima TCu 380A intrauterine contraceptive device (Farla Medical Ltd) |  |  | IU device |
| 49127 | Copper T380 A intrauterine contraceptive device (R.F. Medical Supplies Ltd) |  |  | IU device |
| 50899 | Novaplus T 380 Ag intrauterine contraceptive device normal (R.F. Medical Supplies Ltd) |  |  | IU device |
| 51443 | Novaplus T 380 Cu intrauterine contraceptive device normal (R.F. Medical Supplies Ltd) |  |  | IU device |
| 55209 | Novaplus T 380 Cu intrauterine contraceptive device mini (R.F. Medical Supplies Ltd) |  |  | IU device |
| 56568 | Novaplus T 380 Ag intrauterine contraceptive device maxi (R.F. Medical Supplies Ltd) |  |  | IU device |
| 58198 | Novaplus T 380 Ag intrauterine contraceptive device mini (R.F. Medical Supplies Ltd) |  |  | IU device |
| 66415 | Intrauterine contraceptive device |  |  | IU device |
| 67254 | Mirena 20micrograms/24hours intrauterine device (Dowelhurst Ltd) |  |  | IU system |
| 2819 | Norplant 228mg Implant (Hoechst Marion Roussel) | 7030202 | Parenteral Progestogen-Only Contraceptives | Implant |
| 9592 | Implanon 68mg implant (Organon Laboratories Ltd) |  |  | Implant |
| 16624 | Levonorgestrel 228mg Implant |  |  | Implant |
| 2748 | Mirena 20micrograms/24hours intrauterine device (Bayer Plc) | 06040103/07030203 | Progestogen Products Doubling (In House Use)/ Intra-Uterine Progestogen-Only Device | IU system |
| 6906 | Levonorgestrel 20micrograms/24hours intrauterine device |  |  | IU device |
| 57359 | Mirena 20micrograms/24hours intrauterine device (Mawdsley-Brooks & Company Ltd) |  |  | IU system |
| 60564 | Levonorgestrel 13.5mg intrauterine device |  |  | IU device |
| 60632 | Jaydess 13.5mg intrauterine device (Bayer Plc) |  |  | IU device |
| 66047 | Levosert 20micrograms/24hours intrauterine device (Gedeon Richter (UK) Ltd) |  |  | IU device |
| 72106 | Kyleena 19.5mg intrauterine device (Bayer Plc) |  |  | IU device |
| 73046 | Levonorgestrel 19.5mg intrauterine device |  |  | IU device |
| 13209 | Etonogestrel 68mg implant |  |  | implant |
| 44196 | Nexplanon 68mg implant (Merck Sharp & Dohme Ltd) |  |  | implant |
| 938 | NovaT | Other |  | IU device |
| 2747 | multiload |  |  | IU device |
| 4904 | gyne-t |  |  | IU device |
| 5265 | multiload |  |  | IU device |
| 5405 | NovaT |  |  | IU device |
| 5647 | t-safe |  |  | IU device |
| 14038 | flexi-t |  |  | IU device |
| 14158 | multiload |  |  | IU device |
| 14295 | flexi-t |  |  | IU device |
| 14959 | tt380 |  |  | IU device |
| 18826 | multisafe |  |  | IU device |
| 19143 | gynefix |  |  | IU device |
| 21686 | gynefix |  |  | IU device |
| 26488 | neosafe |  |  | IU device |
| 33367 | load 375 |  |  | IU device |
| 36984 | mini tt380 |  |  | IU device |
| 36995 | ut380 |  |  | IU device |
| 38015 | ut380 |  |  | IU device |
| 43404 | steriload |  |  | IU device |
| 45739 | optima |  |  | IU device |
