# Supplementary material for: The acceptability of asking women to delay removal of a long-acting reversible contraceptive to take part in a preconception weight loss programme: a mixed methods study using qualitative and routine data (Plan-it)
Source: BMC Pregnancy Childbirth. 2022 Oct 18;22:778. doi: 10.1186/s12884-022-05077-0 (PMC9580156; doi:10.1186/s12884-022-05077-0)
Supplement: Supplementary file 2 — Additional file 2. Clinical codes. [file 12884_2022_5077_MOESM2_ESM.docx]

# Additional File 2: Clinical codes

#### (a) Read codes for contraception

| **Med Code** | **Label** |
| --- | --- |
| 11507 | Depot contraception |
| 29297 | Oral contracept. check admin. |
| 29030 | Depot contraceptive - problem |
| 71415 | GMS3 claim - temporary contraceptive (non IUCD) signed |
| 19267 | Oral contraceptive poisoning |
| 19496 | Withdrawal contraception |
| 56806 | [X]Other contraceptive management |
| 98936 | GMS3 claim - temporary contraceptive (non IUCD) paid |
| 4688 | Sheath contraception |
| 25857 | GMS4 claim - contraception (non IUCD) sent to HA |
| 96592 | Contraceptive check first letter |
| 98363 | Contraceptive check third letter |
| 103973 | Migraine induced by oestrogen contraceptive |
| 41407 | Sympto-thermal contraceptn NOS |
| 105984 | Uses contraceptive sponge |
| 91670 | Adv to GP to change pt oral contraceptiv from progestog only |
| 19501 | Oral contraception NOS |
| 19500 | Contraceptive sheath NOS |
| 29034 | Transdermal contraceptive |
| 29958 | [X] Adverse reaction to unspecified oral contraceptive |
| 46842 | GMS4 claim - contraception (non IUCD) due next visit |
| 94002 | Contraceptive registration |
| 16887 | Oral contraceptive claim |
| 100878 | Stopped using contracep sponge |
| 29035 | Uses sympto-thermal contracepn |
| 32976 | Hypertension induced by oral contraceptive pill |
| 5836 | Pill-oral contraceptive claim |
| 22936 | Contraceptive diaphragm |
| 60409 | [V]Contraceptive cream prescription |
| 72028 | [V]Contraceptive foam fitting |
| 6413 | [V]Contraceptive cap fitting |
| 3940 | Adverse reaction to unspecified oral contraceptive |
| 20716 | [V]Repeat prescription of oral contraceptive |
| 15806 | Post-coital contraception NOS |
| 22947 | Depot contraception stopped |
| 22940 | Depot contraceptive-no problem |
| 12989 | Contraceptn from other agency |
| 14830 | Contraceptive sheath |
| 19499 | Oral contraceptive started |
| 29033 | Sympto-thermal contraception |
| 106646 | Emergency contraception indicated |
| 97329 | GMS4 claim - contraception (IUCD) due with new IUCD |
| 57146 | Contraceptive sponge |
| 90951 | Advice to GP to change pt oral contraceptive from combined |
| 27523 | Headache caused by oral contraceptive pill |
| 69159 | Contraceptive sponge failure |
| 180 | Oral contraceptive prescribed |
| 22935 | Post-coital contraception NOS |
| 19508 | Oral contraceptive re-started |
| 13005 | Diaphragm contraception |
| 6255 | CAP contraception |
| 22941 | Spermicide alone contraception |
| 13003 | Depot contraceptive NOS |
| 11810 | FP1001 - contraception claim |
| 8387 | [V]Oral contraceptive prescription |
| 72078 | GMS3 claim - temporary contraceptive (IUCD) sent to HA |
| 12993 | Oral contraceptive repeat |
| 20581 | Oral contraception - problem |
| 106129 | Contraceptive sponge NOS |
| 22938 | Depot contraceptive repeated |
| 90120 | GMS4 claim - contraception (non IUCD) paid |
| 103361 | Contraceptive sheath problem |
| 30766 | Contraceptive usage NOS |
| 17879 | [V]Repeat prescription of oral contraceptive |
| 102867 | Problem with contraception |
| 29032 | Spermicidal contraceptive |
| 6759 | Post-coital contraception |
| 41 | Contraception |
| 19506 | Depot contraceptive given |
| 110589 | Barrier contraception method |
| 6586 | Contraceptive claims |
| 71434 | GMS3 claim - temporary contraceptive (IUCD) signed |
| 13007 | Oral contraception -no problem |
| 20354 | Combined oral contraceptive |
| 61591 | GMS4 claim - contraception (IUCD) signed |
| 102367 | Uses contraception |
| 47020 | Contraceptive sheath issued |
| 69241 | GMS4 claim - contraception (non IUCD) due |
| 113582 | GMS4 claim - contraception (IUCD) paid |
| 5666 | Oral contraception |
| 12995 | Oral contraceptive |
| 100565 | GMS4 claim - contraception (non IUCD) forgot to claim |
| 8538 | Emergency contraception |
| 5839 | Contraceptive administration |
| 13006 | Contracep. NOS - other agency |
| 12992 | Depot contraceptive |
| 19507 | Uses contraceptive sheath |
| 41761 | GMS3 claim - temporary contraceptive (non IUCD) sent to HA |
| 18538 | Pill contraceptive admin |
| 17291 | Oral contraceptive admin |
| 104174 | Progestogen only oral contraceptive |
| 19505 | Mini-pill: oral contraceptive |
| 37428 | [V]Repeat prescription of oral contraceptive (OC) |
| 9252 | Missed contraceptive pill |
| 20360 | Oral contraceptive changed |
| 25858 | GMS4 claim - contraception (non IUCD) signed |
| 96063 | GMS4 claim - contraception (non IUCD) up to date |
| 98074 | Contraceptive check second letter |
| 100069 | GMS4 claim - contraception (non IUCD) returned unpaid |
| 68735 | GMS4 claim - contraception (IUCD) sent to HA |
| 6358 | Pill - oral contraception |
| 22944 | Spermicide + sheath contracep. |
| 39563 | Stopped using sheath |
| 22943 | Uses sheath + spermicide |
| 100655 | Uses contr sponge & spermicide |
| 2438 | Progestagen only oral contrac. |
| 94215 | Neuroleptic depot injection |
| 12994 | Progestagen only pill |
| 20388 | Adverse reaction to combined oestrogens and progestogens |
| 715 | 'Morning after' pills given |
| 15255 | Oral contraceptive stopped |
| 102608 | UK medical eligib criteria for contraceptive use 2009 cat 2 |
| 1848 | Contraception counselling |
| 2446 | Contraception contraindicated |
| 102610 | UK medical eligib criteria for contraceptive use 2009 cat 3 |
| 96591 | Contraceptive check invitation |
| 109059 | Combined oral contraceptive pill contraindicated |
| 107774 | Emergency contraception declined |
| 102678 | Education for contraceptive sheath |
| 63613 | Contraceptive scheme card issued |
| 102676 | Education for postcoital contraceptive |
| 3618 | General contraceptive advice |
| 103240 | Education for contraceptive diaphragm |
| 6477 | Emergency contraception advice |
| 102957 | UK medical eligib criteria for contraceptive use 2009 cat 4 |
| 95989 | Discussion about contraception injection |
| 47069 | [V]Unspecified contraceptive management |
| 12711 | [V]Contraceptive management |
| 102604 | Planned contraception method |
| 52109 | [V]Surveillance previously prescribed contraceptive methods |
| 102609 | UK medical eligib criteria for contraceptive use 2009 cat 1 |
| 107837 | GMS4 claim - contraception (non IUCD) cancelled |
| 102521 | Education for transdermal contraceptive patch |
| 108120 | Referral to contraception and sexual health service |
| 109280 | Progestogen only oral contraceptive contraindicated |
| 100652 | Contraceptive advice for patients with epilepsy |
| 19497 | Rhythm method contraception |
| 104432 | Education about missed dose of oral contraceptive |
| 26212 | Advice about progestogen only oral contraceptive |
| 101143 | Contraceptiv advice for patients with epilepsy not indicated |
| 105422 | Discussion about risks of combined oral contraception |
| 110889 | Natural contraception |
| 108552 | Education for spermicidal contraceptive |
| 2282 | Oral contraceptive advice |
| 102190 | Contraceptive advice for patients with epilepsy declined |
| 100549 | Parental consent for contraceptive treatment |
| 26039 | [V]Other specified contraceptive management |
| 102876 | UK medical eligib criteria for contraceptive use 2009 risk |
| 269 | Total abdominal hysterectomy NEC |
| 813 | Abdominal hysterectomy and bilateral salpingoophorectomy |
| 873 | Vaginal hysterectomy |
| 1729 | Subtotal abdominal hysterectomy |
| 1830 | Abdominal hysterectomy and right salpingoopherectomy |
| 2058 | Abdominal hysterectomy and left salpingoopherectomy |
| 2448 | Abdominal hysterectomy |
| 3064 | Wertheim hysterectomy |
| 3433 | TAH - Tot abdom hysterectomy and BSO - bilat salpingophorect |
| 3666 | Hysterectomy NEC |
| 6231 | H/O: hysterectomy |
| 7411 | Vaginal hysterectomy NEC |
| 7798 | TAH - total abdom hysterectomy & bilateral salpingoophorect |
| 7949 | Abdominal hysterectomy and left salpingoophorectomy |
| 10888 | Post hysterectomy vaginal vault prolapse |
| 11662 | Abdominal hysterectomy with conservation of ovaries |
| 12910 | No smear – hysterectomy |
| 12920 | No smear - benign hysterectomy |
| 18980 | Laparoscopic hysterectomy |
| 19088 | Laparoscopic vaginal hysterectomy |
| 19182 | Radical hysterectomy |
| 23863 | Abdominal hysterectomy & bilateral salpingoophorectomy (BSO) |
| 31312 | Abdominal hysterectomy & excision of periuterine tissue NEC |
| 42949 | Vaginal hysterectomy with conservation of ovaries |
| 47215 | Ward vaginal hysterectomy |
| 49408 | Cervical smear to continue post hysterectomy |
| 52057 | Schauta radical vaginal hysterectomy |
| 54109 | Vaginal hysterectomy and excision of periuterine tissue NEC |
| 69607 | Bonney abdominal hysterectomy |
| 94490 | Total abdominal hysterectomy with conservation of ovaries |
| 94549 | Laparoscopic subtotal hysterectomy |
| 94934 | Subtotal abdominal hysterectomy with conservation of ovaries |
| 97020 | Lap assist vag hysterectomy with bilat salpingo-oophorectomy |
| 100097 | Heaney vaginal hysterectomy |
| 109060 | Subtotl abdominal hysterectomy & bilat salpingo-oophorectomy |
| 109193 | Vaginal hysterectomy and right salpingo-oophorectomy |
| 109229 | Subtotal abdominal hysterectomy & left salpingo-oophorectomy |
| 109286 | Radical hysterectomy with bilateral salpingo-oophorectomy |
| 109351 | Vaginal hysterectomy and left salpingo-oophorectomy |
| 109686 | Subtotl abdominal hysterectomy & right salpingo-oophorectomy |
| 111335 | Radical hysterectomy with conservation of ovaries |
| 168 | [V]Sterilisation |
| 2932 | [V]Post-sterilisation vasoplasty or tuboplasty |
| 3532 | H/O: sterilisation – female |
| 4178 | Laparoscopic bilateral female sterilisation |
| 6338 | [V]Admission for sterilisation |
| 6841 | Open bilateral female sterilisation |
| 6847 | Endoscopic bilateral female sterilisation |
| 7163 | Other open female sterilisation |
| 7903 | Other endoscopic female sterilisation |
| 8231 | [V]Other sterilisation |
| 12998 | Contraception: female sterilis |
| 18751 | Other laparoscopic female sterilisation |
| 43832 | Sterilising procedure |
| 48256 | [V]Reattempted sterilisation |
| 60089 | [V]Post-sterilisation tuboplasty |
| 5210 | H/O: tubal ligation |
| 9724 | [V]Admission for tubal ligation |
| 14653 | Open bilateral ligation of fallopian tubes |
| 35984 | Pomeroy open bilateral ligation of fallopian tubes |
| 55932 | Open ligation of remaining solitary fallopian tube |

#### (b) Read codes for menopause

| **MedCode** | **Label** |
| --- | --- |
| 38792 | Hormone replacement therapy bleed pattern - normal |
| 62292 | Hormone replacement therapy bleed pattern - not relevant |
| 26606 | Hormone Replacement Therapy ongoing treatment |
| 49111 | Hormone replacement therapy bleed pattern - abnormal |
| 59447 | Hormone replacement therapy bleed pattern - no bleeding |
| 13054 | Health education - hormone replacement therapy |
| 1671 | Hormone replacement therapy |
| 337 | Hormone replacement therapy |
| 12611 | Hormone replacement therapy requested |
| 11923 | Hormone replacement therapy review |
| 50119 | Years on hormone replacement therapy |
| 52904 | [X]Other specified menopausal and perimenopausal disorders |
| 38395 | Postmenopausal osteoporosis with pathological fracture |
| 2087 | Premature menopause NOS |
| 45409 | Menopausal and postmenopausal disorder NOS |
| 9171 | Menopausal and postmenopausal disorders |
| 19954 | Artificial menopause state |
| 1583 | Postmenopausal bleeding |
| 36514 | Menopause: LH, FSH checked |
| 67495 | Post menopausal urethritis |
| 25549 | Menopausal concentration lack |
| 22074 | Menopause follow-up assessment |
| 86026 | Menopausal profile |
| 21464 | Menopause monitoring NOS |
| 17628 | Postmenopausal disorders |
| 707 | Postmenopausal atrophic vaginitis |
| 30359 | Perimenopausal atrophic vaginitis |
| 20628 | Menopause initial assessment |
| 17442 | Menopause symptoms present |
| 2702 | Menopause: bone density check |
| 4462 | H/O: post-menopausal bleeding |
| 93526 | Perimenopausal menorrhagia |
| 15436 | Post menopausal atrophic urethritis |
| 828 | Menopausal symptoms NOS |
| 94499 | Early menopause |
| 9700 | Postmenopausal osteoporosis |
| 15283 | Menopausal sleeplessness |
| 4043 | Menopausal or female climacteric state |
| 30590 | Postmenopausal state |
| 4383 | Menopause |
| 9313 | Menopause monitoring |
| 9547 | Menopausal flushing |
| 62797 | Menopause: dietary advice |
| 17051 | Menopausal arthritis |
| 46997 | Postmenopausal postcoital bleeding |
| 814 | Hot flushes - menopausal |
| 15022 | Premenopausal menorrhagia |
| 28046 | Other menopausal and postmenopausal states |
| 58681 | Menopause: sexual advice |
| 18730 | Menopausal headache |
| 21534 | Menopause: gen counselling |
| 52129 | H/O: hormone replacement (HRT) |
| 38723 | HRT: combined oestrog/progest |
| 34489 | HRT contraindicated |
| 33436 | HRT: unopposed oestrogen |
| 29275 | HRT changed |
| 26608 | HRT side-effects |
| 26607 | HRT started |
| 25078 | HRT stopped |
| 13199 | Hormone implant - HRT |
| 13198 | HRT prophylaxis |
| 73849 | [X] Adverse reaction to clonidine |
| 67676 | Clonidine poisoning |
| 26107 | Adverse reaction to clonidine |
| 11438 | Hot flushes |

#### (c) Read codes for planning, trying and difficult to get pregnancy

| **MedCode** | **Label** |
| --- | --- |
| 102359 | Pregnancy advice for patients with epilepsy |
| 5778 | Pregnancy advice |
| 36903 | Pregnancy advice NOS |
| 12996 | Trying to conceive |
| 10205 | Advice relating to pregnancy and fertility |
| 2949 | Seen in fertility clinic |
| 4571 | Seen in fertility clinic |
| 26088 | [V]Infertility investigation and testing |
| 9036 | Fertility counselling |
| 9983 | Treatment for infertility NOS |
| 5239 | Referral to fertility clinic |
| 33458 | Female infertility therapy |
| 1810 | Treatment for infertility |
| 9133 | Fertility investigation of female NEC |
| 2548 | Procreat/fertility counselling |
| 39295 | [V]Infertility general advice and counselling |
| 1154 | Infertility investigations NOS |
| 36458 | Other female infertility |
| 102589 | Infertility care |
| 16131 | Infertility investigation -fem |
| 41692 | Female infertility test abnormal |
| 53018 | Other female infertility NOS |
| 17756 | Female infertility test normal |
| 7246 | Subfertility |
| 25361 | Fertility counselling |
| 10445 | Advice on fertility and infertility |
| 7351 | Fertility problem |
| 33510 | [V]Procreative management |
| 100920 | Preconception advice for patients with epilepsy |
| 41319 | Preconception care |
| 41033 | EDC - Estimated date of conception |
| 63344 | Estimated date of conception |
| 25307 | Reproductive counselling |
| 20847 | [V]IVF |
| 93810 | Other specified in vitro fertilisation (IVF) |
| 10238 | IVF |
| 52626 | In vitro fertilisation (IVF) |
| 102280 | IVF with pre-implantation for genetic diagnosis |
| 90936 | In vitro fertilisation (IVF) NOS |
| 89966 | IVF with donor sperm |
| 57000 | IVF with intracytoplasmic sperm injection (ICSI) |
| 21532 | Folic acid advice - pre-pregnancy |
| 49884 | Diabetic pre-pregnancy counselling |
| 4609 | Pre-pregnancy counselling |
| 50937 | Referral to diabetes preconception counselling clinic |
| 102767 | Preconception advice for diabetes mellitus |
| 10761 | Preconception advice |
| 69751 | [V]Unspecified infertility management |
| 2957 | Infertility problem |
| 1808 | Infertility - female |
| 37047 | A/N care: H/O infertility |
| 40072 | Infertility studies |
| 30392 | Female infertility NOS |
| 26150 | [V]Other specified infertility management |
|  |  |
| 9938 | [V]Infertility management |
| 64063 | IVF with donor eggs |
| 97981 | IVF intracytoplasmic sperm injection (ICSI) and donor egg |
| 89716 | IVF with surrogacy |
| 20977 | Adverse reaction to clomiphene |
| 73091 | Maternity grant advice |
| 459 | Endoscopic bilateral occlusion of fallopian tubes NOS |
| 1891 | Endoscopic bilateral occlusion of fallopian tubes |
| 4935 | Open bilateral occlusion of fallopian tubes NOS |
| 17760 | Other endoscopic occlusion of fallopian tube |
| 23346 | Occlusion of vagina |
| 28161 | Endoscopic occlusion of left fallopian tube |
| 32187 | Open bilateral occlusion of fallopian tubes |
| 42543 | Unilateral occlusion of fallopian tube |
| 45721 | Other open occlusion of fallopian tube |
| 50204 | Endoscopic occlusion of right fallopian tube |
| 50773 | Other specified open bilateral occlusion of fallopian tubes |
| 55972 | Other open occlusion of fallopian tube NOS |
| 58048 | Endoscopic occlusion of remaining solitary fallopian tube |
| 59338 | Other specified endoscopic occlusion of fallopian tube |
| 61157 | Endoscopic bilateral occlusion of fallopian tubes OS |
| 64187 | Endoscopic occlusion of fallopian tube NOS |
| 68272 | Endoscopic unilateral occlusion of fallopian tubes |
| 68614 | Occlusion of cervix |
| 69724 | Other specified other open occlusion of fallopian tube |
| 95677 | In vitro fertil with pre-implantation for genetic diagnosis |
| 97196 | Ant scr shows homozygot/compound heterozygote of genetic sig |
| 97802 | Ant scr show homozygot/compound heterozygote no genetic sign |
| 100785 | Anten screen, partner tested and no genetic risk identified |
| 102280 | IVF with pre-implantation for genetic diagnosis |

#### (d) Read codes for planned and unplanned pregnancy

| **MedCode** | **Label** | **Planned/unplanned** |
| --- | --- | --- |
| 14877 | Pregnant - ? planned | Planned |
| 20240 | Pregnant - planned | Planned |
| 30365 | Wanted pregnancy | Planned |
| 8767 | Open reversal of female sterilisation | Planned |
| 8844 | Open reversal of female sterilisation | Planned |
| 24538 | Endoscopic reversal of female sterilisation | Planned |
| 24657 | [V]Reversal of sterilisation | Planned |
| 40709 | Open reversal of female sterilisation NOS | Planned |
| 41343 | Laparoscopic reversal of female sterilisation | Planned |
| 56297 | Other specified endoscopic reversal of female sterilisation | Planned |
| 66719 | Endoscopic reversal of female sterilisation NOS | Planned |
| 97970 | Other specified open reversal of female sterilisation | Planned |
| 29915 | Open reversal of tubal ligation | Planned |
| 6197 | [V]Failed sterilisation NOS | Unplanned |
| 37019 | Female sterilisation failure | Unplanned |
| 15338 | Pregnancy unplanned ? wanted | Unplanned |
| 7517 | Unplanned pregnancy | Unplanned |
| 30618 | Unplanned pregnancy | Unplanned |
| 14842 | Pregnant - unplanned - wanted | Unplanned |
| 15567 | Pregnant -unplanned-not wanted | Unplanned |
| 23421 | IUD failure - pregnant | Unplanned |
| 29692 | Pregnant, IUD failure | Unplanned |
| 20623 | [V]Problems related to unwanted pregnancy | Unplanned |
| 5044 | Unwanted pregnancy | Unplanned |
| 15033 | [V]Other unwanted pregnancy | Unplanned |
| 50421 | Unwanted pregnancy | Unplanned |
| 32975 | Pregnant, diaphragm failure | Unplanned |
| 14994 | Pregnant, sheath failure | Unplanned |
| 40851 | Depot contraceptive failure | Unplanned |
| 19503 | Progestogen-only Pill failure | Unplanned |
