# Supplementary material for: The acceptability of asking women to delay removal of a long-acting reversible contraceptive to take part in a preconception weight loss programme: a mixed methods study using qualitative and routine data (Plan-it)
Source: BMC Pregnancy Childbirth. 2022 Oct 18;22:778. doi: 10.1186/s12884-022-05077-0 (PMC9580156; doi:10.1186/s12884-022-05077-0)
Supplement: Supplementary file 3 — Additional file 3. Assumptions agreed by the Plan-it study team in advance. [file 12884_2022_5077_MOESM3_ESM.docx]

Additional File 3. Assumptions agreed by the Plan-it study team in advance:

- For those with a pregnancy, only codes that were within 456 days (1 year + 3 months) prior to the pregnancy start date were investigated. For those with no pregnancy, only codes that were within 456 days of the LARC event were investigated.
- If the gap between a LARC removal and LARC situ events was less than 28 days, it was assumed that the patient was having a LARC check-up. These two events were combined as one rather than include twice.
- When the LARC in-situ/insertion and LARC removal event on the same date, LARC replacement was assumed and the LARC removal event was excluded.
- For clinical codes that appeared between a pregnancy start and end, only the planned pregnancy and unplanned pregnancy code were included.
- LARC in situ or insertion within a week of a pregnancy start was coded as unplanned pregnancy.
- When the first LARC code occurs between a pregnancy start and end and after a week of a pregnancy start, the whole event was excluded.
- When both unplanned and planned pregnancy code occur between LARC removal and pregnancy start, the code that was closest to the pregnancy start was used as an indicator for grouping the woman.
- For a patient who has both alternative contraception and trying/difficult to get pregnant code on the same day, the trying/difficult to get pregnant code was used as an indicator and the contraception code excluded.
- Patients with too many planned/unplanned codes were coded as Unable to code.
