# Supplementary material for: The acceptability of asking women to delay removal of a long-acting reversible contraceptive to take part in a preconception weight loss programme: a mixed methods study using qualitative and routine data (Plan-it)
Source: BMC Pregnancy Childbirth. 2022 Oct 18;22:778. doi: 10.1186/s12884-022-05077-0 (PMC9580156; doi:10.1186/s12884-022-05077-0)
Supplement: Supplementary file 4 — Additional file 4. Participant flow diagram. [file 12884_2022_5077_MOESM4_ESM.docx]

**Additional File 4. Participant flow diagram.**

**A. Study population – women of reproductive age**

**C. Pregnancy events**

**B. LARC events**

**Pre-defined study population (n=318,443)**

All patients extracted from CPRD data
(n=20,520,012)

586,698 LARC product codes from 413,528 patients

2,217,406 LARC medication codes from 689,699 patients

340,640 pregnancy events from 113,262 women

Exclude 245,935 events from 56,625 women§

16,394 events from 15,297 women

Exclude 7,679 events from 7,029 women§§

24,073 events from 22,326 women

**315,755 women with 929,099 LARC events of which 15,297 women have at least one pregnancy**

Exclude 70,632 events from 34,311 women††

Exclude 52,564 codes and 35,735 patients‡

Exclude 551,042 codes and 120,019 patients†

Exclude patients*
n=17,887,141

534,134 LARC product codes from 377,793 women

1,666,364 LARC medication codes within 569,680 women

Number of women at reproductive age (n=2,632,871)

94,705 events from 56,637 women

2,200,498 LARC events from 624,782 women

Exclude women not in the study population n=2,314,831

Exclude 1,198,541 events and 306,742 women**

**Final study population n=318,040**

1,001,957 LARC events from 318,040 women

Exclude 72,858 events and 2,285 women‡‡

929,099 LARC events from 315,755 women

**Exclusions**

| **CPRD data*** | **LARC Medication codes**† | **LARC Product codes**‡ | **Pregnancy registry**§ |
| --- | --- | --- | --- |
| •Male or indeterminate or unknown gender (9,806,591)  •Patient CPRD date not up-to-standard (1,406,355)  •Year of birth < 1961 or > 2002 (3,895,933)  •Died before 2009 (25,917)  •Patient registered with practice after 2018 (48,068)  •Patient current period of registration with the practice after 2018 (8,941)  •Patient transferred out the practice before 2009 (2,229,378)  •Practice last data collection date ended before 2009 (95,446)  •Practice up-to-standard date begin after 2018 (63,904)  •With less than 1-year data available (88,242)  •Not met the criteria in the specified years (218,366) | •Male (37,147 codes from 113,919 patients)  •Year of birth < 1961 or > 2002 (421,288 codes from 5,918 patients)  •Code with no event date (1,839)  •Same code on the same date (90,768) | •Male (356 codes from 169 patients)  •Year of birth < 1961 or > 2002 (4,8511 codes from 35,566 patients)  •Same code on the same date (3,697) | •Exclude Pregnancy <2009 or >2018 (232,410 events)  •Exclude events that were duplicated or < 31 days or overlapped events (13,525 events) |
| **Merged LARC medication and LARC product codes**** | **Pregnancy registry in study population**†† | | |
| •Women not in the pre-defined study population (867,870 codes)  •Event < 2009 or >2018 (330,671 codes) | •Pregnancy events belongs to women with no LARC event (235)  •First pregnancy event before the first LARC (62,160)  •First LARC code between pregnancy start and end (3,625) •Multiple pregnancy events after one LARC event (4,847) | | |
| **LARC code and CPRD data**‡‡ | **LARC code and pregnancy registry**§§ | | |
| •Excluded LARC event happened before age 16 or after age 48 (8,482 codes within 1,927 women)  •Excluded LARC removal codes that are ≤ 28 days between the next LARC in-situ or LARC insertion code (LARC replacement) (62,026 codes)  •Excluded LARC code in between pregnancy start and end (2,350 codes) | •Excluded pregnancy events that were 1 year + 3 month after the LARC events (7,679 events within 7,029 women) | | |
| **Study population:** Women of reproductive age (16-48) with a LARC event between 01/01/2009 and 31/12/2018 | | | |
