# Supplementary material for: The acceptability of asking women to delay removal of a long-acting reversible contraceptive to take part in a preconception weight loss programme: a mixed methods study using qualitative and routine data (Plan-it)
Source: BMC Pregnancy Childbirth. 2022 Oct 18;22:778. doi: 10.1186/s12884-022-05077-0 (PMC9580156; doi:10.1186/s12884-022-05077-0)
Supplement: Supplementary file 5 — Additional file 5. Defining the groups based on LARC use, events related to planning a pregnancy andcontrary events, and conception. [file 12884_2022_5077_MOESM5_ESM.docx]

**Additional File 5. Defining the groups based on LARC use, events related to planning a pregnancy and contrary events, and conception**

| **Study Classification** | **LARC event** | **Read code** | **Pregnancy observed** | **Number of scenarios** | **Number of women** |
| --- | --- | --- | --- | --- | --- |
| **Planning a pregnancy** | Any (removal/ inserted/in situ) | A read code to indicate the pregnancy was being planned (planned pregnancy code or trying/difficult to get pregnant) either between a LARC (removal/inserted/in situ) and pregnancy start, or between a pregnancy start and end. | Yes | 1,635 | 1,616 |
|  |  | A read code to indicate a pregnancy was being planned (planned pregnancy code or trying/difficult to get pregnant). | No | 4,871 | 4,717 |
| **Possibly planning a pregnancy** | Any | No read code to indicate the pregnancy was planned (planned pregnancy code or trying or difficult to get pregnant) or not planning a pregnancy (alternative contraception and menopause). No read code to indicate pregnancy was unplanned (unplanned pregnancy code). | Yes | 10,902 | 10,387 |
| **Probably not planning** | Any | A read code to indicate a pregnancy was not being planned (alternative contraception). | Yes | 3,851 | 3,761 |
|  | Inserted/in situ | No code present to indicate that a LARC was removed or was planning/not planning a pregnancy (alternative contraception, menopause). | No | 379,495 | 277,144 |
| **Not enough information** | Removal | After a LARC removal code, no read code to indicate that the woman was planning/not planning a pregnancy. | No | 73,290 | 69,455 |
| **Not enough information** | N/A | Unable to classify due to unclear coding. | N/A | 65 | 65 |
